# Orientation Experiences and Navigation Aid Use: A Self-Report Lifespan Study on the Role of Age and Visuospatial Factors

**DOI:** 10.3390/ijerph19031225

**Published:** 2022-01-22

**Authors:** Veronica Muffato, Erika Borella, Francesca Pazzaglia, Chiara Meneghetti

**Affiliations:** 1 Department of General Psychology, University of Padova, 35131 Padua, Italy; erika.borella@unipd.it (E.B.); francesca.pazzaglia@unipd.it (F.P.); chiara.meneghetti@unipd.it (C.M.); 2 Interuniversity Research Center in Environmental Psychology (CIRPA), 00185 Rome, Italy

**Keywords:** spatial behaviors, visuospatial working memory, navigation, sense of direction, spatial anxiety, GPS, aging, lifespan

## Abstract

Spatial orientation is essential for daily life, but it deteriorates with aging. The present study was aimed at investigating age changes across the adult lifespan in the self-reported use of navigation aids and everyday orientation experiences, as well as investigating to what extent these are related to visuospatial working memory (VSWM) and self-reported wayfinding inclinations. A sample of 456 people aged 25–84 years rated how much they use navigation aids (maps, GPS, verbal directions), how much they went out, and how much they reached or lost their way to unfamiliar destinations (in 2016). Then, they performed the jigsaw puzzle test (VSWM) and questionnaires on sense of direction, pleasure in exploring, and spatial anxiety. The results showed that increasing age is related to a lower tendency to go out, fewer experiences of finding one’s way and getting lost, a lower level of GPS use, and increased verbal directions use. After age changes were accounted for, VSWM was related to aid use and orientation experiences (except for losing one’s way), wayfinding inclinations (especially spatial anxiety) to using a map, and orientation experiences. Overall, other than age, VSWM and one’s wayfinding attitudes can play a role–albeit it a modest one–in spatial behaviors.

## 1. Introduction

The abilities to navigate and orient oneself to the environment are essential in daily life for one to successfully reach destinations and avoid getting lost. Such abilities have been shown to decline with increasing age [1,2]. These impairments can limit older people’s independence and safety of living, thus having an impact on their perceived quality of life [3,4]. Therefore, within the healthy and active aging domain perspective, it is important to extend our theoretical knowledge on older people’s navigation issues and on the factors that can be related to them.

Most of the current knowledge about the navigation and aging issue derives from studies asking people to learn unfamiliar paths from navigation, a sensorimotor experience in which information is acquired from the person’s point of view [5]. The environment learning process and the related mental representation of it (or, the cognitive map [6,7]) can be assessed using several spatial recall tasks. Research has shown a decline with aging in landmark knowledge, location knowledge, and path knowledge (see [2] for a review). At the same time, asking people to self-report their experienced navigation issues is a modality used to detect everyday habits related to navigation-based experiences [8]. Studies investigating the navigation issues in older adults both with self-reports (in terms of the strategies adopted while navigating, as well as the characteristics of the environment that are believed to increase the experience of getting lost) and new environment learning showed a link between subjective–self-reported–and objective–performance-based–modalities [9]. Thus, self-reports–shown to be related to objective navigation learning–could be informative regarding people’s navigation performance in everyday life. Indeed, moving in the environment and reaching destinations are spatial behaviors that are important for navigation [10] and merit being investigated across the adult lifespan for better analyzing the navigation issue (age-related changes and their extent) as experienced in daily life.

### 1.1. Spatial Behaviors across the Adult Lifespan

#### 1.1.1. Everyday Orientation Experiences

Previous research on the self-reported everyday spatial behaviors of people across the adult lifespan showed reduced self-reported mobility (e.g., trips per week and average driving frequency) with increasing age [11]. People also report less walking time with increasing aging, especially over the age of 75 years [12,13]. Older adults reported preferring trips to well-known places with respect to unfamiliar places [14]. They reported preferring less complex trips (e.g., involving only one destination at a time rather than multiple destinations), and using public transportation less [15]. These spatial behaviors and experiences could reflect older adults’ tendency to minimize the experience of getting lost [8,16]. For instance, it was shown that the fear of disorientation prevented older people from using public buildings [17]. However, due to these navigation issues, older people tend to restrict the activities in which they are involved, which, in turn, constitutes a barrier for healthy and active aging.

#### 1.1.2. Use of Navigation Aids

Among spatial behaviors across the adult lifespan, it is of interest to examine not only the everyday orientation experiences (going out, reaching places, and/or experiences of getting lost) but also the use of navigation aids. They comprise the instruments (e.g., maps, global positioning system-GPS) and modalities (e.g., asking for verbal directions) used to support moving in the environment. Navigation aid use is of interest for the identification of people’s spatial profiles [18] and, related to this, for people’s navigation performance accuracy and navigation habits. Indeed, in young adults, navigation aid use relates to the quality of the mental representation of the environment [7], which, in turn, helps with successful navigation. Maps have been shown to support the construction of refined spatial mental representations [19,20]. Their use has a positive impact on recall task performance involving reading them before navigating in the new environment [21,22], as well as reading them during navigating [23]. Thus, maps are an effective tool for navigation performance accuracy. In contrast, to date, the use of GPS (navigator) seems not to facilitate the construction of cognitive maps and navigation performance [24,25], and its use seems to be a distraction from one’s surroundings [19,24,25,26]. Ishikawa and colleagues [27]) found that participants who used GPS traveled longer distances, stopped more often during their navigation experiences, and had lower levels of the recall of the environment learned compared with those who used maps or had no aids. Subsequent research confirmed GPS use’s detrimental in spatial mental representations. Another modality used when navigating, then, involves asking for verbal directions [28]. Mental representations derived from verbal descriptions have spatial properties [29]. The use of verbal directions in navigation has been shown to favor the recall of spatial information when they convey information about the environment’s global layout (e.g., describing how landmarks are located in relation to one another using cardinal points) and not when landmarks are presented only as reference points in navigation [30,31]. Overall, these studies showed that the presence of maps and verbal directions-presenting global information about the environment seems to support individuals’ navigation accuracy. Meanwhile, the use of GPS seems not to support navigation.

However, from the little evidence available for the elderly, with increasing age, the role of navigation aids seems to have changed its relevance. Research showed a decrease in map learning with aging [32] and learning a route from a map before did not consequently help with the path navigation process [33]. Also, having a map available while navigating did not increase the navigation performance. However, older adults felt more secure when the map was there [34]. Concerning GPS use, it seems to positively support navigation. When older adults are trained to use GPS tools, they increase their successful driving (e.g., reaching the destination) [35]. Furthermore, older adults reported using GPS to plan their trips to minimize their self-reported fear of getting lost [8]. On the contrary, spatial verbal directions appeared not to support spatial mental representations of older adults compared with other inputs, such as maps [36,37,38].

Overall, aging changes people’s spatial behaviors, such as their mobility [11], experiences of reaching places or getting lost [8,15], and the use of navigation aids [8,34]. Such navigation-based spatial behaviors could relate to individual factors other than age, however.

### 1.2. The Relationship between Individual Factors and Spatial Behaviors

Previous research focused mainly on lab-based environment learning performance and suggested that people can differ widely in their navigation capabilities and spatial behaviors due to individual factors, such as visuospatial abilities [39,40]. In adulthood, route learning is related to individual spatial abilities, such as visuospatial working memory (VSWM), which is the ability to retain and process visuospatial information [41]. This pattern of results has also been found in the adult lifespan, from youth to older age [42]. Despite the decline in this ability with aging [43,44], it still supports older adults’ environment learning from navigation [45].

At the same time, other than spatial abilities, environment learning performance relates to self-reported wayfinding inclinations, i.e., individual preferences and attitudes related to wayfinding [46,47] that define one’s spatial profile [48]. These include-among others–people’s self-reported sense of direction [49,50], individuals’ preferences for representing environments [49,50], exploration tendency and pleasure in doing so [48,51], and spatial anxiety [52]. Altogether higher ratings (and ratings lower in spatial anxiety) can be called positive wayfinding inclinations [18]. Although related to each other, these wayfinding inclinations contribute differently to route learning as a function of the spatial knowledge tested in young adults [47]. In studies adopting an adult lifespan perspective, self-reported wayfinding inclinations, such as sense of direction and pleasure in exploring, tend to remain stable throughout the adult lifespan, whereas spatial anxiety is susceptible to increasing in later life (over 70 years old; see [53,54]). Wayfinding inclinations (such as a greater sense of direction, greater pleasure in exploring places, a lower level of spatial anxiety) support environment knowledge (such as pointing cardinal points from their positions [51]) and the repetition of a route previously learned. Here, the evidence of self-reported spatial behaviors in daily life with an individual’s spatial abilities and wayfinding attitudes in both young and older adults is scarce. An expectation is set forth by the Meneghetti et al. [18] study that showed that the self-reported use of maps as navigation aids in young adults is related to positive wayfinding inclinations but not to spatial abilities. Similarly, Münzer et al. [20] found that older adults who self-report using maps as navigation aids also report a higher positive wayfinding inclination in terms of strategies. However, no studies have adopted an adult lifespan perspective to examine these issues.

### 1.3. Rationale and Aims

The present study started from the premise that self-reported spatial behaviors could be informative of people’s navigation difficulties related to their objective spatial abilities [8,9]. The literature showed age changes-namely increasing age from youth to older age-in self-reported orientation experiences (such as mobility [11] and the experiences of reaching places or getting lost [8,15]) and in the use of navigation aids (such as maps [34] or GPS [8]). Apart from age, an adequate spatial profile [18,48] in terms of spatial abilities (VSWM) and inclinations could support functional spatial behaviors. However, little evidence regarding the young has suggested a relation between self-reported spatial behaviors in everyday life (use of navigation aids) and individual visuospatial factors [18,20]. This issue merits being investigated with an adult lifespan perspective to better capture any age-related changes. In fact, in the adult lifespan, evidence exists that environment-based self-reports are linked, even if differently, to spatial performance [51], and individual visuospatial factors are related to environmental performance [47]. It is plausible that self-reported spatial behaviors (people-orientation experiences and navigation aid use) might also be linked to individual visuospatial factors. This issue has yet to be investigated. Examining age, visuospatial abilities, and people’s attitudes in their relationship to spatial behavior contribute to a better understanding of older adults’ everyday mobility. The self-reported experience could be informative about the impact of navigation issues on the daily life of older adults, to promote healthy and active aging.

In this context, the present study was aimed at investigating from an adult lifespan perspective the relationship among self-reported everyday spatial behaviors, such as orientation experiences (tendency to go out, successfully reaching an unfamiliar destination, or getting lost), people’s use of navigation aids (maps, GPS, verbal directions), individual visuospatial factors in terms of VSWM, and one’s wayfinding inclinations (sense of direction, pleasure in exploring places, and spatial anxiety).

#### Hypothesis

In considering people’s self-reported experiences of going out and orienting themselves in the environment, we hypothesized–in line with previous literature-that increasing age could be associated with a tendency to go out less [11], and with experiences of getting lost, given that older adults tend to avoid situations that might be disorienting [8]. We explored whether people also report successfully reaching their destinations less frequently as they grow older. To our knowledge, no studies have investigated this issue; however, successful wayfinding is important for navigation [10].

In addition to age, we newly investigated the role of visuospatial abilities (VSWM) and wayfinding inclinations. We hypothesized that a better VSWM and positive wayfinding inclinations (such as a stronger sense of direction, pleasure in exploring, and a lower level of spatial anxiety) could be related to a higher frequency of going out, successfully reaching destinations and fewer experiences of getting lost due to better navigation performance [51]. Moreover, given previous evidence suggesting that people can use support (such as maps and/or GPS) to minimize their negative orientation experiences [8], such as reaching places/getting lost, we also explored–as a complementary aim–whether navigation aids predict a decrease in getting lost and an increase in successfully reaching destinations.

The present study is the first, to our knowledge, to explore age-related changes in the use of navigation aids. Given that older adults make less use of technology, in Italy at least [55], we expected increasing age to be associated with lower usage of GPS, but not of maps or verbal directions. As well as age, we also explored how older people’s VSWM and wayfinding inclinations related to their use of navigation aids. Given previous evidence, the only expectation was a relationship between map use and positive wayfinding inclinations [18,20]. As for the use of GPS and verbal directions, and their relationship with VSWM and wayfinding inclinations, as no previous research is available, we envisaged two possible scenarios. People with good spatial profiles, in terms of their VSWM and wayfinding inclinations, could rely less on the use of external support, or else they might use them more because they add to their enjoyment of their orientation experiences.

To investigate the relationship between spatial behaviors and age, VSWM and attitudes, we asked a sample of people 25–84 years old to self-report their everyday orientation experiences (how much they go out, how often they reach or lose their way to unfamiliar destinations, and their use of navigation aids such as maps, GPS, verbal directions). They also performed a VSWM test and completed questionnaires on their wayfinding inclinations (sense of direction, pleasure in exploring, and spatial anxiety). All this information is presented in the following section, followed by the results regarding everyday orientation experiences and the use of navigation aids, and a discussion of our findings.

## 2. Materials and Methods

In the following sections, we will present the participants involved in the study (sample size and sample characteristics), the materials used (the questionnaire measuring self-reported spatial behaviors, VSWM test, and wayfinding questionnaires), and then the procedure.

### 2.1. Participants

The study involved 456 healthy adults, 152 young people 25–44 years old (*M* = 33.02, *SD* = 5.89), 152 middle-aged people 45–64 years old (*M* = 54.55, *SD* = 5.17), and 152 older people 65–84 years old (*M* = 73.14, *SD* = 5.79). A power analysis run with the “pwr” library in R for linear models and considering 7 coefficients in the models (see below), showed that 121 people were needed to obtain a power of 0.80, an effect size of 0.15, and a *p* of 0.01. Therefore, we considered at least 121 people per group. For including older adults with normal cognitive functioning, an inclusion criterion of Montreal Cognitive Assessing (MoCA [56]) score of at least 22 were needed for participants aged 60 or older (see [57] for the Italian normative sample) (*M* MoCA = 27.17, *SD* = 1.68). None of the participants, then, had a history of psychiatric, neurological, or other diseases capable of causing cognitive, visual, auditory, or motor impairments [58] and all lived independently, as assessed by a sociodemographic questionnaire [59]. All participants had attended the compulsory schooling level (years of education: young: *M* = 14.73, *SD* = 2.85; middle-aged: *M* = 12.49, *SD* = 3.16; older adults: *M* = 10.89, *SD* = 3.49), and young adults had more years of education-*F* (2453) = 56.09, η^2^_p_ = 0.20, *p* < 0.01 compared with the middle-aged people (*p* < 0.01), who had more years of education than older people did (*p* < 0.01).

The local ethical committee approved the study, and all participants were informed of its purposes and gave their informed written consent according to the Declaration of Helsinki [60].

### 2.2. Materials

#### 2.2.1. Questions on Everyday Orientation Experiences and Navigation Aid Use 

These are six questions about everyday orientation experiences (three questions) and navigation aid use (three questions) rated on a Likert scale from 1 = not at all to 6 = very much (adapted from [18]). For the everyday orientation experiences, the questions are: (i) “How much do you go out?” (ii) “How often do you successfully reach an unfamiliar destination?” and (iii) “How often do you lose your way in an unfamiliar environment?” For the navigation aid use, the questions were: (iv) “How often do you use a map to orient yourself in a new environment?” (v) “How often do you use a navigator (GPS) to orient yourself in a new environment?” and (vi) “How often do you ask for verbal directions to orient yourself in a new environment?” The rating of each question is considered as a dependent variable in the following analyses. See Appendix A.

#### 2.2.2. Jigsaw Puzzle Test (JPT)

This VSWM task [59] involves mentally recomposing puzzles of objects (from 2 to 10 pieces, i.e., levels of difficulty). Participants must solve at least two of the three puzzles on a given level to proceed to the next one. The final score is the sum of the difficulty levels of the last three puzzles solved (max 29; good test-retest reliability of *r* = 0.83).

#### 2.2.3. Sense of Direction and Spatial Representation Scale (SDSR)

This scale [50,61] measures an individual’s self-assessed sense of direction, the usage of cardinal points, and preferences for the survey- and route-landmark-based mode (13 items). Each item is rated on a Likert scale from 1 (not at all) to 5 (very much), and a single score is calculated by summing all items ([53], max score 65). Cronbach’s α for the present sample was 0.83.

#### 2.2.4. Attitudes towards Orientation Tasks Scale (AtOT)

This scale [61] measures an individual’s pleasure in exploring (e.g., “I like to find new ways in which to reach familiar places”) (10 items). Five of the 10 items are reverse scored, as they express a preference for well-known places and discomfort with unfamiliar ones (e.g., “When I see a new road, I avoid taking it because I don’t know where it goes”). Each item is rated on a Likert scale from 1 (not at all) to 6 (very much), and the sum is calculated (max score 60) after the five negative items are reversed. Cronbach’s α for the present sample was 0.84.

#### 2.2.5. Spatial Anxiety Scale (SA)

This scale [61] measures the degree of anxiety experienced in wayfinding situations (e.g., “Going to an appointment in an unfamiliar part of the city”) (eight items). Each item is rated on a Likert scale from 1 (not at all) to 6 (very much), and the sum is calculated (max score 48). Cronbach’s α for the present sample was 0.87.

### 2.3. Procedure

In an in-presence individual session lasting for 45 min, participants completed a sociodemographic questionnaire, the MoCA (people over 60 years old), the questions about spatial behaviors (frequency of going out, reaching a destination, losing the way, map use, GPS use, verbal directions use), and the JPT (VSWM), SDSR, AtOT, and SA questionnaires in a balanced order.

### 2.4. Data Analysis

Data analysis was performed using R software (R Foundation for Statistical Computing, Vienna, Austria [62]). First with the intent to describe the data, means and standard deviation of all the variables divided by age groups and correlations between all variables in the total sample were computed. Then to accomplish the aim of shedding more light on the effects of the various individual factors on spatial behaviors linear models were run stepwise to determine whether the factors added to each step improved the model (changes in R^2^ were reported). The order in which the factors were inserted into the models reflects the order used in previous spatial cognition studies on young and older adults [45,54] given the subjective self-reported spatial behaviors related to objective navigation learning [9]. Gender and years of education were entered into a baseline model (Step 0) to control for the effect of gender (given its influence in the spatial domain on the young [62], as well on the adult lifespan [53]) and years of education given its role in spatial task performance with increasing age [63]. Then, the age in the continuum was entered to analyze any age changes from 25 to 84 years (Step 1) in self-reported spatial behaviors [8,11]. After these variables were controlled for, the contributions of cognitive abilities, in terms of processing abilities (i.e., VSWM) (as in [45]), and self-reported measures, in terms of wayfinding attitudes (as in [54]), to spatial behaviors were examined. Therefore, in Step 2, JPT performance was inserted as the VSWM measure. Next, in Step 3, self-reported wayfinding inclinations (SDSR, AtOT, and SA) were added to see whether they still had a role after all other factors had been accounted for. For all models, the variance inflation factors did not reveal significant multicollinearity (VIF values ≤ 2.29). 

## 3. Results

Descriptive statistics divided by age groups and correlations between all variables are reported in Table 1.

### 3.1. The Role of Age and Visuospatial Factors in Spatial Behaviors

#### 3.1.1. Everyday Orientation Experiences

For going out (total R^2^ = 0.13), Step 0 accounted for 4% of the variance, with both gender and years of education emerging as a significant predictor. Age (Step 1) emerged as a significant predictor and accounted for another 4% of the variance. JPT (VSWM, Step 2) emerged as a significant predictor, meaning that higher JPT scores are associated with a higher frequency of going out, accounting for 2% of the variance. Step 3 accounted for 3% of the variance, with spatial anxiety emerging as a significant predictor, meaning that a higher level of anxiety is associated with a lower frequency of going out (see Table 2 for the changes in R2, estimates, and *p*-values for all steps).

For the experiences of reaching an unfamiliar place (total R^2^ = 0.12), Step 0 accounted for 2% of the variance, with years of education emerging as a significant predictor. Age (Step 1) emerged as a significant predictor and accounted for 2% of the variance. JPT (VSWM, step 2) emerged as a significant predictor, meaning that higher JPT scores are associated with a higher frequency of successfully reaching unfamiliar places, accounting for 4% of the variance. Step 3 accounted for another 4% of the variance, with spatial anxiety emerging as a significant predictor, meaning that a higher level of anxiety reduces the frequency of reaching an unfamiliar place.

For the experiences of losing one’s way in an unfamiliar place (total R^2^ = 0.22), Step 0 represented 4% of the variance, with years of education emerging as a significant predictor. Age (Step 1) emerged as a significant predictor and accounted for 8% of the variance. JPT (VSWM, Step 2) did not add any variance. Step 3 accounted for another 10% of the variance, with SDSR and spatial anxiety emerging as significant predictors, meaning that a low level of anxiety and a high sense of direction reduces the frequency of getting lost in an unfamiliar place.

To ascertain whether navigation aid use could predict a decrease in getting lost and an increase in successfully reaching one’s destination [8], a further Step 4 was added with the map, GPS, and verbal instruction use as predictors of reaching an unfamiliar place and losing one’s way in an unfamiliar place. For the experience of reaching an unfamiliar place, Step 4 accounted for an additional 2% of the variance, with GPS use emerging as a significant predictor (β = 0.12, 95% CI [0.01, 0.23], *p* = 0.027), meaning that a higher use of GPS is associated with a higher frequency of reaching unfamiliar destinations. No other predictor emerged as significant (maps: β = 0.07, 95% CI [−0.02, 0.17], *p* = 0.130; verbal directions: β = −0.01, 95% CI [−0.10, 0.08], *p* = 0.890). Concerning the experience of losing one’s way in an unfamiliar place, Step 4 did not add any variance, and no predictor emerged as significant (maps: β = 0.05, 95% CI [−0.04, 0.14], *p* = 0.277; GPS: β = −0.02, 95% CI [−0.12, 0.08], *p* = 0.729; verbal directions: β = −0.03, 95% CI [−0.05, 0.12], *p* = 0.468).

#### 3.1.2. Everyday Navigation Aids Use

For the use of the map (total R^2^ = 0.17), Step 0 accounted for 10% of the variance, and years of education emerged as a significant predictor. Age (Step 1) did not emerge as a significant predictor and accounted for 1% of the variance. JPT (VSWM, Step 2) emerged as a significant predictor, meaning that higher JPT scores are associated with the increased use of maps, accounting for 3% of the variance. Step 3 accounted for another 3% of the variance, with AtOT and spatial anxiety emerging as significant predictors, meaning that greater attitudes toward orientation and higher spatial anxiety are associated with higher map use (see Table 2). 

For GPS use (total R^2^ = 0.35), Step 0 accounted for 13% of the variance, with both gender and years of education emerging as significant predictors. Age (Step 1) emerged as a significant predictor and accounted for 19% of the variance. JPT (VSWM, Step 2) emerged as a significant predictor, meaning that higher JPT scores were associated with the increased use of GPS, accounting for 3% of the variance. Step 3 did not account for any variance (see Table 2).

For the use of verbal instructions (total R^2^ = 0.09), Step 0 accounted for 3% of the variance, with gender emerging as a significant predictor. Age (Step 1) emerged as a significant predictor and accounted for 3% of the variance. JPT (VSWM, step 2) emerged as a significant predictor, meaning that high JPT scores were associated with lower use of verbal instructions, accounting for another 3% of the variance. Step 3 did not account for any additional variance (see Table 2).

## 4. Discussion

The present study examined the relationships between several self-reported spatial behaviors and experiences and people’s own spatial ability (as measured by a VSWM task) and wayfinding inclinations (such as sense of direction, pleasure in exploring, and spatial anxiety) in the adult lifespan, from 25 to 84 years old.

Before the main results are presented, the roles of the control variables examined–gender and year of education–are discussed given their crucial role in spatial cognition [62,63]. It is worth noting that both variables emerged in relation to spatial behaviors and experiences. Years of education were related to increased going out, successfully reaching destinations, and the use of maps and GPS. This result confirms that education not only is an important proxy indicator of the cognitive reserve [64,65] and is thus related to active aging but also is an important factor to consider when analyzing older adults’ spatial behaviors [2]. Years of education are also associated with increased experiences of getting lost. This result could reflect that people who go out more and are active in finding their way are inevitably more exposed to potentially negative experiences in the environment, such as getting lost. Taken together, the result for years of education implies the need to promote lifelong learning, even in older age [66], to sustain spatial behaviors and thus navigation in the environment, which, in turn, promotes active aging. Regarding the role of gender, females reported going out less and not using navigation aids, such as maps and GPS, preferring the use of verbal directions. This profile does not help with spatial knowledge acquisition [67], and here, it also seems not to encourage successful spatial behaviors. The female issue in spatial cognition needs to be better investigated [62] altogether with its impact on spatial experiences and behaviors in daily life, especially in aging.

The main interest of the study was to examine the relationship among age, VSWM, and wayfinding inclinations for people (from young to older adults) self-reporting everyday experiences and navigation aid use. The results showed that the individual factors (age, VSWM, wayfinding inclinations) emerged as predictors of the use of navigation aids, self-reported going out, and orientation experiences, with some specificity, existing depending on the dependent variable considered. Each factor in relation to the dependent variables is discussed in the following paragraphs.

Considering the role of age, our results showed the relations-although modest in terms of the variance explained-between age (getting older) and decreasing in going out, decreasing in the frequency of reaching places, and decreasing in the frequency of losing one’s way in unfamiliar environments. These result patterns suggest that aging reflects reduced mobility [11]. Probably going out less is a strategy for avoiding the experience of getting lost [8]. Our results also showed a relationship between increasing age and the lower use of GPS as well as greater use of verbal directions, although it was modest. Concerning GPS use, recent evidence suggested its detrimental role in spatial knowledge acquisition in the young [24]. However, it seems to be a good device for helping older adults to orient to the environment [35]. Therefore, a detailed investigation of its role in the adult lifespan would be of interest for future studies. The use of verbal directions, which is reported to be used more with increased age, is not a good strategy given that studies revealed that it is a less efficient modality for acquiring spatial knowledge compared with, for instance, maps [37]. These results concerning aging and everyday orientation experience highlight the importance of environmental policies promoting age-friendly environments to make older adults feel safe in exploring the environment. Maintaining older adults’ mobility and their ability to find their way in the environment is important for their independence and quality of life [3]. To improve older adults’ navigation skills, training them on efficient orientation strategy use could be another point of interest [68]. Thus, future studies are needed that highlight a procedure for training older adults to rely more on maps and GPS and to rely less on verbal instructions.

However, above the roles of gender, year of education, and age, the role of people VSWM was found to be relevant for all variables except for the experience of losing one’s way (even the relation goes in the same directions of those involving other variables, but it is not significant). Previous research showed the important role of VSWM in supporting spatial knowledge acquisition [47], even in the adult lifespan [42]. The present results expand our knowledge, suggesting that VSWM is also associated with higher, at least self-reported, mobility, greater experiences of success in the environment, and the self-reported functional use of navigation aids (more use of maps, more use of GPS, lower levels of the use of verbal instructions). This pattern of results suggests the importance of maintaining and training the individual VSWM, despite its age changes [69,70,71], given that it represents an ability related to every day successful spatial behaviors and experiences. Regarding the relationship found between VSWM capacity and higher GPS use, this appears to be in contrast with evidence suggesting that GPS may not facilitate the construction of cognitive maps [24,25]. It could be that people with higher spatial competencies are able to use the technology aid functionally and actively. However, these suppositions need to be better clarified in future studies.

Although explaining a little portion of variance, a role of positive and negative wayfinding inclinations in some cases was found, too. Concerning positive wayfinding inclinations, a higher self-reported sense of direction was related to fewer self-reported experiences of losing one’s way in an unfamiliar place. Positive attitudes with regard to experiencing pleasure in exploring places were related to a higher level of the use of maps. Therefore, one’s positive inclinations related to wayfinding in the environment somewhat support experiencing successful experiences in it. In addition, the use of an aid, the map, proved to be functional for navigation [72,73]. On the other hand, having negative wayfinding inclinations, such as a higher level of spatial anxiety, was found to be related to a lower tendency to go out, fewer experiences of reaching unfamiliar destinations, more experiences of getting lost, and greater map use. These results expand the current knowledge about spatial anxiety implied in decreasing spatial knowledge recall in young [30] and older adults [74], also suggesting its detrimental role in people’s spatial behaviors and experiences in daily life. They also call upon the need to consider this aspect as for its applied and clinical value. Note that we found a positive association between spatial anxiety and the use of maps, and we could argue that people who are known to be spatially anxious tend to use a support to orient themselves as a strategy for compensating for their anxiety. The role of spatial anxiety thus also merits further investigation in future studies. However, it seems clear that promoting positive wayfinding inclinations and lowering the level of spatial anxiety in the adult lifespan could be another way in which to maintain positive and successful spatial behaviors and experiences in daily life. It is also worth noting that the role of positive and negative wayfinding inclinations is not found for GPS and verbal directions use, a result that merits further investigation to be confirmed (or not).

Taken together, the results on individual visuospatial factors, such as VSWM and wayfinding inclinations, added insights to individual factors related to self-reported spatial behaviors, such as orientation experiences and navigation aid use. Implications can be seen across the adult lifespan, particularly in the view of healthy and active aging. The knowledge of spatial navigation impairments with increasing age is here expanded considering self-reported spatial behaviors and experiences. Therefore, understanding the lived experiences of people can provide insight that can be then used to improve their navigation abilities [8]. Considering the role of individual factors–given the heterogeneity of the aging process–seems to be a way in which to deeply understand the aging navigation issue to counteract–or slow down–age changes and to avoid its impact on people’s perceived quality of life [3,4]. Implications could also be applied at the policy level. Transportation systems in cities should consider older individuals’ mobility needs [75], for instance adopting technology as a facilitator for favoring orientation in the environment (e.g., readable maps) [76] and training people, especially older ones, in its use.

However, some limitations of the present study should be mentioned as well. First, the variance that the factors considered explain is limited. This means that our factors of interest played a part, but other factors play a role in predicting people’s spatial behaviors and should be investigated as well (e.g., personality factors, the role of the characteristic of the environment [77]). Another limitation is that self-assessed spatial behavior, although informative of everyday life, could not be precise and could not completely reflect the actual spatial behaviors that people have. Indeed, despite the evidence of the relationship between self-reported spatial behaviors with environmental performance in older adults [9], this does not guarantee an overlapping of the results obtained with subjective evaluations and objective ones. Future studies should corroborate people’s subjective views of their everyday spatial behaviors with objective assessments, such as navigation performance in both familiar and unfamiliar environments. Another limitation of the present study is that it does not explicitly consider the distinction between familiar and unfamiliar environments, as well as where people go (navigation experiences related to a leisure activity, shopping, tourism, visiting relatives, etc.). Indeed, spatial behaviors and experiences could be different in well-known or newly explored environments. In aging, although a decline in the spatial learning of new environments occurs, spatial knowledge of one’s city of residence is well preserved [78,79,80]. Future studies should thus investigate spatial behaviors in both familiar and unfamiliar environments. Another limitation is that different Likert scales are used in this study, and this may compromise the reliability of the result since the number of rating bars in the Likert scale is sensitive to the outcome of the measurement [81]. This issue merits future investigation. Finally, given the importance of individual differences, longitudinal studies could add more information to everyday spatial behaviors in aging [82]. For example, data for the present study were collected in 2016, and it would be interesting to update them to investigate any changes in people’s spatial behaviors.

## 5. Conclusions

In conclusion, the present study sheds light on people’s self-assessed spatial behaviors in relation to individual differences, adopting an adult lifespan perspective. Considering the role of increasing age, associated with unsuccessful spatial behaviors, the role of spatial abilities (VSWM) and positive wayfinding inclinations played a role in promoting spatial behaviors. On the other hand, the role of spatial anxiety is detrimental for spatial behaviors, especially for losing in an unfamiliar place. For promoting active and healthy aging with regard to maintaining people’s mobility and active use of navigation aids, it seems to be important to consider people’s spatial competencies and wayfinding inclinations from youth to older age.

### Future Directions and Recommendations

Considering the limitations and the results of the present study, future directions could be focused on efforts to promote healthy and active aging. Several individual factors relating to their spatial behaviors should be considered and thoroughly investigated in future studies to support older people’s mobility and interest in exploring the environment. For example, their use of GPS for navigation should be analyzed in-depth, given our increased reliance on GPS, even in aging [24,35], and the mixed findings on its influence on young and older adults’ navigation accuracy [35]. Regarding the various individual differences relating to successful spatial behaviors, future studies could examine how to improve older adults’ everyday spatial behaviors, by giving them training on the efficient use of orientation strategies (such as maps and GPS), basic cognitive abilities (such as VSWM), and on metacognitive reflection of their wayfinding inclinations, for instance.

## Figures and Tables

**Table 1:** Means and standard deviation of variables in the three age groups and correlations among variables.

	25–44 Years Old M (SD)	45–64 Years Old M (SD)	65–84 Years Old M (SD)	1.	2.	3.	4.	5.	6.	7.	8.	9.	10.	11.
1. Age	33.03 (5.89)	54.55 (5.17)	73.14 (5.79)	-										
2. Years of education	14.73 (2.85)	12.49 (3.15)	10.89 (3.49)	−0.43	-									
3. Jigsaw Puzzle Test (VSWM)	21.88 (4.67)	18.61 (5.20)	13.94 (5.11)	−0.59	0.40	-								
4. Sense of direction and spatial representation (SDSR)	37.60 (7.96)	37.64 (8.42)	37.45 (8.71)	−0.01	0.17	0.19	-							
5. Attitudes toward Orientation Experiences (AtOT)	34.05 (8.44)	33.58 (8.67)	29.32 (9.37)	−0.21	0.25	0.37	0.59	-						
6. Spatial anxiety (SA)	22.21 (6.83)	22.57 (7.33)	23.68 (7.33)	0.09	−0.11	−0.24	−0.38	−0.60	-					
7. Map use	3.37 (1.79)	3.27 (1.75)	2.57 (1.51)	−0.20	0.31	0.31	0.20	0.29	−0.10	-				
8. GPS use	4.32 (1.62)	3.26 (1.92)	1.90 (1.48)	−0.53	0.32	0.47	0.08	0.24	−0.18	0.28	-			
9. Verbal directions use	3.19 (1.35)	3.56 (1.37)	3.76 (1.52)	0.16	0.02	−0.23	−0.07	−0.09	0.13	0.05	−0.16	-		
10. Going out	4.89 (1.08)	4.61 (1.12)	4.18 (1.25)	−0.26	0.18	0.27	0.18	0.23	−0.25	0.18	0.24	0.05	-	
11. Experiences of reaching unfamiliar destinations	5.06 (1.14)	4.88 (1.35)	4.57 (1.49)	−0.17	0.13	0.28	0.12	0.20	−0.27	0.17	0.24	−0.08	0.19	-
12. Experiences of getting lost in unfamiliar places	3.66 (1.49)	2.72 (1.54)	2.44 (1.30)	−0.33	0.19	0.14	−0.22	−0.18	0.24	0.07	0.15	0.01	0.04	0.02

Note: Given multiple comparisons, only *p*s < 0.05/13 = 0.004 were considered as significant. Correlations with *p* < 0.004 in bold type.

**Table 2:** Regression models for everyday orientation experiences (going out, the experience of reaching an unfamiliar place, the experience of losing one’s way in an unfamiliar place) and navigation aid use (map, GPS, and verbal directions use).

	**Going Out**				**Experiences of Reaching an Unfamiliar Place**				**Experiences of Losing one’s Way in an Unfamiliar Place**				**Map Use**				**GPS Use**				**Verbal Directions Use**			
	**ΔR^2^**	**β**	**CI**	***p***	**ΔR^2^**	**β**	**CI**	***p***	**ΔR^2^**	**β**	**CI**	***p***	**ΔR^2^**	**β**	**CI**	***p***	**ΔR^2^**	**β**	**CI**	***p***	**ΔR^2^**	**β**	**CI**	***p***
Step 0 (baseline)	0.04				0.02				0.04				0.10				0.13				0.03			
Gender		−0.19	[−0.37, −0.01]	0.041		−0.17	[−0.35, 0.02]	0.075		0.10	[−0.08, 0.28]	0.275		−0.18	[−0.36, −0.01]	0.042		−0.34	[−0.52, −0.17]	<0.001		0.31	[0.13, 0.49]	0.001
Years of education		0.18	[0.09, 0.27]	<0.001		0.13	[0.04, 0.22]	0.007		0.19	[0.10, 0.28]	<0.001		0.30	[0.22, 0.39]	<0.001		0.32	[0.23, 0.40]	<0.001		0.02	[−0.07, 0.12]	0.600
Step 1 (age)	0.04				0.02				0.08				0.01				0.19				0.03			
Age		−0.22	[−0.32, −0.12]	<0.001		−0.14	[−0.24, −0.04]	0.007		−0.31	[−0.41, −0.22]	<0.001		−0.09	[−0.19, 0.01]	0.073		−0.49	[−0.57, −0.40]	<0.001		0.21	[0.11, 0.31]	<0.001
Step 2 (objective measure)	0.02				0.04				0.00				0.03				0.03				0.03			
JPT		0.15	[0.03, 0.26]	0.011		0.26	[0.15, 0.38]	<0.001		−0.10	[−0.22, 0.01]	0.068		0.23	[0.12, 0.34]	<0.001		0.19	[0.10, 0.29]	<0.001		−0.20	[−0.32, −0.09]	0.001
Step 3 (subjective measures)	0.03				0.04				0.10				0.03				0.00				0.00			
SDSR		0.09	[−0.02, 0.20	0.110		0.01	[−0.10, 0.12]	0.833		−0.13	[−0.24, −0.03]	0.014		0.05	[−0.06, 0.16]	0.379		−0.05	[−0.15, 0.04]	0.277		−0.02	[−0.13, 0.09]	0.721
AtOT		−0.00	[−0.13, 0.13]	0.971		−0.03	[−0.16, 0.10]	0.689		−0.12	[−0.25, 0.00]	0.051		0.22	[0.09, 0.34]	0.001		0.03	[−0.09, 0.14]	0.662		0.07	[−0.06, 0.21]	0.284
SA		−0.17	[−0.28, −0.06]	0.002		−0.23	[−0.34, −0.12]	<0.001		0.18	[0.07, 0.28]	0.001		0.11	[0.01, 0.22]	0.035		−0.06	[−0.15, 0.04]	0.241		0.09	[−0.02, 0.20]	0.117
R^2^	0.13				0.12				0.22				0.17				0.35				0.09			

*Note*. SDSR = Sense of Direction and Spatial Representation; AtOT = Attitude toward Orientation Tasks; SA = Spatial Anxiety; JPT = Jigsaw Puzzle Test (VSWM).

## Data Availability

The data presented in this study are openly available in FigShare at https://doi.org/10.6084/m9.figshare.17306939.v1 (accessed on 21 December 2021).

## Appendix A

Questions on everyday orientation experiences and navigation aid use (adapted from [18])

Please rate the following spatial behaviors on a Likert scale, from 1 = not at all to 6 = very much/often, considering your everyday habits:

(1) How much do you go out? 1 2 3 4 5 6
(2) How often do you successfully reach an unfamiliar destination? 1 2 3 4 5 6
(3) How often do you lose your way in an unfamiliar environment? 1 2 3 4 5 6
(4) How often do you use a map to orient yourself in a new environment? 1 2 3 4 5 6
(5) How often do you use a navigator (GPS) to orient yourself in a new environment? 1 2 3 4 5 6
(6) How often do you ask for verbal directions to orient yourself in a new environment? 1 2 3 4 5 6
